# Low-intensity microcurrent therapy promotes regeneration of atrophied calf muscles in immobilized rabbits

**DOI:** 10.7555/JBR.32.20180056

**Published:** 2018-08-20

**Authors:** Gi Young Park, Dong Rak Kwon, Yong Suk Moon

**Affiliations:** 1. Department of Rehabilitation Medicine, Muscle Research Center, Catholic University of Daegu School of Medicine, Daegu 42472, Republic of Korea; 2. Department of Anatomy, Catholic University of Daegu School of Medicine, Daegu 42472, Republic of Korea.

**Keywords:** microcurrent, intensity, atrophy, muscle, cast

## Abstract

The purpose of this study was to investigate the intensity-specific regenerative effects of microcurrent therapy on gastrocnemius muscle atrophy induced by cast-immobilization in rabbits. Fifteen rabbits were randomly allocated to 3 groups after cast removal: cast-immobilization and sham microcurrent therapy for 2 weeks (group 1); cast-immobilization and microcurrent therapy (25 μA) for 2 weeks (group 2); cast-immobilization and microcurrent therapy (5,000 μA) for 2 weeks (group 3). Clinical parameters [calf circumference, compound muscle action potential (CMAP) of the tibial nerve, thickness of gastrocnemius muscle], cross sectional area of gastrocnemius muscle fibres, and immunohistochemistry was evaluated. The clinical parameters representing mean atrophic changes in group 2 were significantly lower than those in group 3. The cross sectional area of the gastrocnemius muscle fibres and immunohistochemical parameters in group 2 were significantly greater than those in group 3. The results showed that low-intensity microcurrent therapy can more effectively promote regeneration in atrophied gastrocnemius muscle than high-intensity microcurrent therapy.

## Introduction

Skeletal muscle dysfunction and atrophy limit quality of life and daily living activities in patients apart from the underlying disease conditions^[1]^. Deconditioning and reduced muscle activity are estimated to be the most relevant factors, which lead to muscle atrophy and loss of function in patients^[1–2]^.

In particular, treating soft tissue and bone injuries by immobilisation may result in skeletal muscle atrophy and decreased functional performance^[3–4]^. Immobilisation causes significant muscle remodelling including loss of myofibrillar proteins, changes in metabolic enzyme activities, and vascular and neural alterations^[5]^. The rapid loss in myofibrillar proteins during immobilisation is induced by a transient decrease in protein synthesis, followed by increased protein degradation, resulting in net protein loss^[6]^. Therefore, regardless of the cause of muscle atrophy, effective skeletal muscle recovery appears to be dependent on protein synthesis.

In addition, myogenic precursor cells (satellite cells), which are activated by muscle injury, will undergo cell division^[7]^, then repair and regenerate adult skeletal muscle tissues^[8–10]^. However, the proliferative potential of satellite cells in injured skeletal muscle may be facilitated or impaired as the consequence of extracellular stimuli, such as mechanical stress and immobilisation^[11–12]^.

Microcurrent therapy (MT) involves therapeutic application of very low electric current (<1 mA), which is usually sub-sensory for the body. A previous study demonstrated that MT might prevent progression of GCM muscle atrophy and facilitate the regeneration of muscle cells^[13]^. These effects could increase protein synthesis and stimulate satellite cell proliferation. Therefore, MT has the potential to become an effective therapeutic intervention to recover muscle atrophy induced by chronic immobilization.

The intensity of MT plays an important role in the effective treatment of muscle damage. According to the previous studies, when an electric current with an intensity of 100–500 μA was applied to treat muscle damage, the healing process including amino acid transport, triphosphate generation, and protein synthesis. Then healing effect increased by 30%–40% above the control level along with the activation of myogenic precursor cells (satellite cells)^[14–16]^. On the contrary, when the intensity exceeded 1,000 μA, these bio-stimulatory effects were reversed. A previous study using 25 μA to treat muscle atrophy showed good regeneration *in vivo* and *in vitro* study^[13]^. Other studies reported healing effect of high intensity electrical stimulation in disused muscle atrophy^[17–18]^. In one study, the intensity of stimulation was strong enough to cause palpable and visible muscle contraction. Given the paucity of data and uncertainty about the effective intensity in treatment, we aimed to investigate the regenerative effects of different intensities of MT on gastrocnemius (GCM) muscle atrophy induced by IC in rabbits.

## Materials and methods

### Animals and grouping

The study protocol was approved by the Institutional Animal Care and Use Committee of our institution. All experimental procedures were carried out in accordance with the guidelines for the care and use of laboratory animals set by the Institutional Animal Care and Use Committee of our institution.

Fifteen male New Zealand White rabbits, aged 12 weeks with an average weight of 3.3 kg (2.8–3.6 kg) were used. The rabbits were randomly allocated to 3 groups by computerized random numbers: group 1: IC for 2 weeks and sham MT for 2 weeks after cast removal (CR); group 2: IC for 2 weeks and MT (25 μA) for 2 weeks after CR; group 3: IC for 2 weeks and MT (5,000 μA, capable of inducing palpable and visible muscle contraction) for 2 weeks after CR. The appearance of the sham MT was identical to the real stimulator with no electrical current.

### Immobilisation by cast (IC)

The right GCM muscle was immobilized by cast for 2 weeks. According to the standardised IC procedure, the right knee and ankle of the rabbit were placed in an extended position using a splint made of PVC-plastic, a non-adhesive bandage and an adhesive elastic bandage (Tensoplast^®^; Smith & Nephew Medical, London, UK)^[16]^. Following the IC, muscle samples from the GCM muscle of the right hind limbs were taken for microscopic evaluation. The left limbs were not used as controls, because unilateral hind limb immobilisation could cause loading on the contralateral side. All surgical procedures were performed under general anaesthesia using intramuscular injections, each containing a dose of 8 mg/kg (body-weight) of xylazine (Rompun^®^; Bayer Co., Seoul, Korea) and 30 mg/kg (body-weight) of ketamine (Ketar^®^; Yuhan Co., Seoul, Korea).

### Microcurrent therapy (MT)

Hind limb-hair epilation was performed on all rabbits with a commercial hair remover. The MT generator was programmed to provide an alternating current characterised by a monophasic rectangular pulse format with polarity reversal every 2 seconds. For each rabbit, the electrical patches for MT were placed onto the skin over the GCM muscle proximally (anode) as well as distally (cathode). The GCM muscle of the rabbits in group 2 and 3 underwent a 60-minute microcurrent stimulation daily for 2 weeks, under anaesthesia with ketamine and xylazine. In group 1, the GCM muscle of the rabbits was stimulated with sham MT, under anaesthesia with ketamine and xylazine. The rabbits were allowed to ambulate freely in the cage during the other time. During MT, no muscle contraction was observed in the hind limb of the rabbits.

### Clinical parameters

The circumference of calf area, compound muscle action potential (CMAP) of the tibial nerve, and thickness of medial GCM muscle were measured by ultrasound prior to euthanasia of the rabbits. All parameters were measured by a physiatrist who was blinded to the group allocation throughout the study. At the time of study, the physiatrist had 13 years’ experience of performing musculoskeletal ultrasound and 20 years’ of performing electrophysiological study. For ultrasound measurements, the ultrasound probe was placed parallel to the muscle fibres to avoid anisotropic artefact^[19]^. For measuring the medial GCM muscle thickness and calf circumference, the knee was flexed at 90 degrees to keep the ankles relaxed. Longitudinal ultrasound image was obtained at a fixed point on the medial surface of GCM muscle, which is in the middle of two reference points (one point was located at the proximal one-third of a longitudinal line from midpoint between both malleoli of ankle to midpoint between both femoral epicondyles; the other point was located at the medial end on a transverse line perpendicular to the point on the longitudinal line). The thickness of medial GCM muscle was measured from the superficial to the deep fasciae in real-time ultrasound. The largest calf circumference was measured using a tape. The active recording electrode was placed on the midpoint surface of the GCM muscle, and the reference electrode was placed on the subcutaneous tissue of the ankle. The tibial nerve was electrically stimulated at the popliteal fossa, and the highest CMAPs were recorded after 8–10 supramaximal stimuli.

Atrophic changes in right calf circumference, CMAP of the tibial nerve, and thickness of GCM muscle fibres were estimated using the following equation: [Lt. side - Rt. side/Lt. side × 100].

### Tissue preparation

The rabbits were sacrificed under general anaesthesia after all intramuscular injections. The medial and later GCM muscle fibres of all rabbits were segmented and fixed using neutral buffered formalin for 24 hours. The specimens were embedded in paraffin (Paraplast; Oxford, St. Louis, MO, USA) and sliced transversally into 5-μm thick serial sections.

### Immunohistochemistry

Muscle sections were immunohistochemically stained for type I fibers using monoclonal anti-myosin antibody (skeletal, slow; Sigma-Aldrich, St. Louis, MO, USA). And sections were immunostained for markers of proliferating satellite cells using monoclonal anti-proliferating cell nuclear antigen (PCNA) antibody (PC10; Santa Cruz Technologies, Dallas, TX, USA) and monoclonal anti-bromodeoxyuridine (BrdU) antibody (Clone BU-33; Sigma-Aldrich). To accomplish BrdU staining, all subject rabbits were injected with 25 mg/kg (body-weight) of BrdU (B5002; Sigma-Aldrich) intraperitoneally. Twenty four hours after BrdU administration, the rabbits were sacrificed and paraffin embedded sections were prepared. The sections were incubated in 0.1% trypsin for 10 minutes at 37 °C and 1 N HCl for 30 minutes at 56 °C to denature the DNA. All sections for immunohistochemistry were washed with phosphate buffered saline (PBS). Endogenous peroxidases were inactivated by pre-incubation in 0.3% H_2_O_2_ in PBS for 30 minutes, and non-specific protein binding was blocked in PBS containing 10% normal horse serum (Vector Laboratories, Burlingame, CA, USA) for 30 minutes. The sections were incubated in primary antibodies (1:100–1:500) for 2 hours at room temperature and washed 3 times with PBS. The secondary antibody (biotinylated anti-mouse IgG, 1:100; Vector Laboratories) was placed on the muscle sections for 1 hour at room temperature and washed 3 times with PBS. The avidin-biotin-peroxidase complex (ABC;Vector Laboratories) was placed on the sections for 1 hour and washed 3 times with PBS, followed by a peroxidase reaction obtained by using 0.05 mol/L Tris-HCl (pH 7.6) containing 0.01% H_2_O_2_ and 0.05% 3,3'-diaminobenzidine (DAB; Sigma-Aldrich). The sections were then counterstained with hematoxylin and mounted. The slides were examined with Axiophot Photomicroscope (Carl Zeiss, Oberkochen, Germany) and AxioCam MRc5 (Carl Zeiss).

### Histomorphometric analysis

A previous study reported that type 1 muscle fibre was predominantly affected in immobilization-induced GCM atrophy than type 2^[13]^. Therefore, type 1 muscle fibres were analysed in the current study. The sections were immunohistochemically stained for type 1 muscle fibres using monoclonal anti-myosin antibodies (skeletal, slow; Sigma-Aldrich). All histologic analyses were performed by an anatomist who was blinded to the group allocation throughout the study. At the time of study, the anatomist had 20 years’ experience of performing histologic examination. The sections were examined with the Axiophot Photomicroscope (Carl Zeiss) and the images of 5 randomly selected fields from each group were captured with the AxioCam MRc5 (Carl Zeiss). The entire muscle cross section was determined from digital images (×100) of the anti-myosin immunostained muscle sections. The cross sectional area (CSA) of the anti-myosin positive type 1 muscle fibre was traced using the image morphometry program (AxioVision SE64; Carl Zeiss), and the average CSA of the type 1 muscle fibre was measured.

### Evaluation of immunohistochemical staining

The anti-PCNA or anti-BrdU immunostaining slides were examined with AxioCam MRc5 (Carl Zeiss) interfaced with an Axiophot Photomicroscope (Carl Zeiss). Twenty randomly selected fields from each group were photographed and the AxioVision SE64 (Carl Zeiss) program was used for analysis. Within each image, the number of anti-PCNA or anti-BrdU positive cells or nuclei and the total number of muscle fibres was counted. The PCNA or BrdU ratio was analyzed as the ratio of the number of anti-PCNA/anti-BrdU positive cells or nuclei to the 1,000 muscle fibres.

### Statistical analysis

An initial power analysis based on the pilot results determined that 15 subjects yielded a power of 0.8 at a significance level of 0.05. Statistical analyses were performed using the SPSS program for windows version 19.0 (SPSS Inc., Chicago, IL, USA). In addition to standard descriptive statistical calculations (means and standard deviations), ANOVA was used to determine intra- and inter-group statistical differences. When ANOVA yielded results indicating significant difference between the groups, a Tukey’s test was performed. The mean values were followed by 95% confidence intervals, and all the data were expressed as the means±standard deviations. The statistically significant levels were pre-determined at *P*<0.05. The post-hoc power analysis was performed, and the power was estimated to be>0.95.

## Results

**Fig.1:**
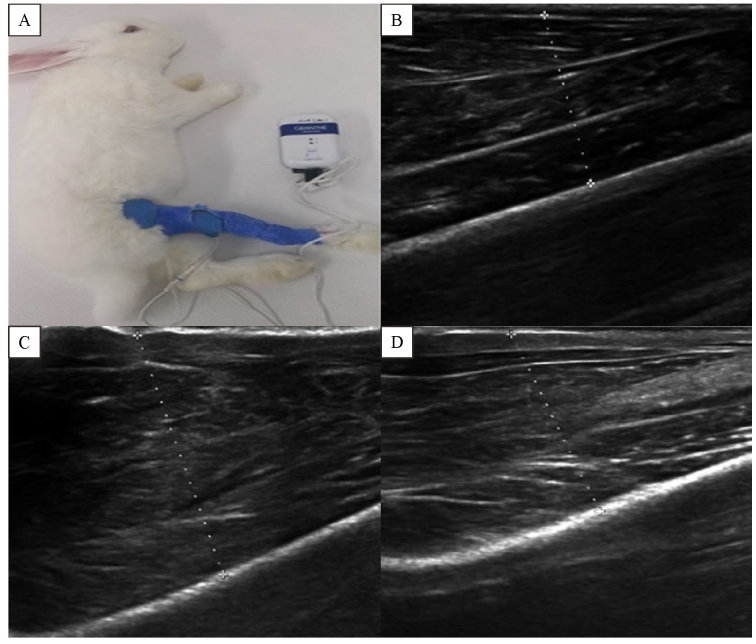
**Cast immobilization in a rabbit model under microcurrent therapy and ultrasound images of right gastrocnemius muscles. ** A: Rabbits were immobilized by cast and treated with microcurrent. Microcurrent electrode pads were placed on right posterior aspect of the calf and right lateral thigh. B: Severe atrophy was seen in the right gastrocnemius muscle after 2 weeks of immobilization by cast and sham microcurrent therapy for 2 weeks after cast removal. C: Regenerated right gastrocnemius muscle was seen after 2 weeks of immobilization by cast and microcurrent therapy (25 μA) for 2 weeks after cast removal. D: Regenerated right gastrocnemius muscle was seen after 2 weeks of immobilization by cast and microcurrent therapy (5,000 μA) for 2 weeks after cast removal.

There were significant differences among the clinical, imaging, and electrophysiologic parameters in the three groups ( *P*<0.05, ***Table 1***). The mean atrophic changes (%) in right calf circumference ( ***Fig. 1***), amplitude of CMAP of the right tibial nerve, and right GCM muscle thickness in group 2 (15.28±1.55, 13.40±0.45, 13.30±0.43) and group 3 (18.16±0.40, 14.10±0.27, 14.10±0.59) were significantly lower than those in group 1 (33.68±1.09, 30.90±1.01, 32.98±1.44), respectively ( *P*<0.05, ***Table 1***). The mean atrophic changes of right calf circumference, amplitude of CMAP of the right tibial nerve, and right GCM muscle thickness in group 2 were significantly smaller than those in group 3 ( *P*<0.05, ***Table 1***). Parameters in group 2 were the smallest degree of mean atrophic changes among the three groups ( *P*<0.05, ***Table 1***).

**Tab.1:** Comparison of clinical parameters among three groups

Group		Atrophic change (%)		
		Rt. calf muscle circumference	CMAP on Rt. tibial nerve	Rt. GCM muscle thickness on US
Group 1 ( *n*=5)		33.68±1.09 ^a)^	30.90±1.01^ a)^	32.98±1.44^ a)^
Group 2 ( *n*=5)		15.28±1.55 ^b)^	13.40±0.45^ b)^	13.30±0.43^ b)^
Group 3 ( *n*=5)		18.16±0.40 ^c)^	14.10±0.27^ c)^	14.10±0.59^ c)^

Values are presented mean±standard error. Group 1: IC for 2 weeks and sham MT for 2 weeks after CR; Group 2: IC for 2 weeks and MT (25 μA for 2 weeks after CR; Group 3: IC for 2 weeks and MT (5,000 μAfor 2 weeks after CR; IC: immobilization by cast; MT: microcurrent therapy; CR: cast removal; CMAP: compound muscle action potential; GCM: gastrocnemius muscle; US: ultrasound; a), b), c): Any two means in the same row with different letters represent a significant difference at *P*<0.05, One Way ANOVA, post-hoc Tukey test.

**Fig.2:**
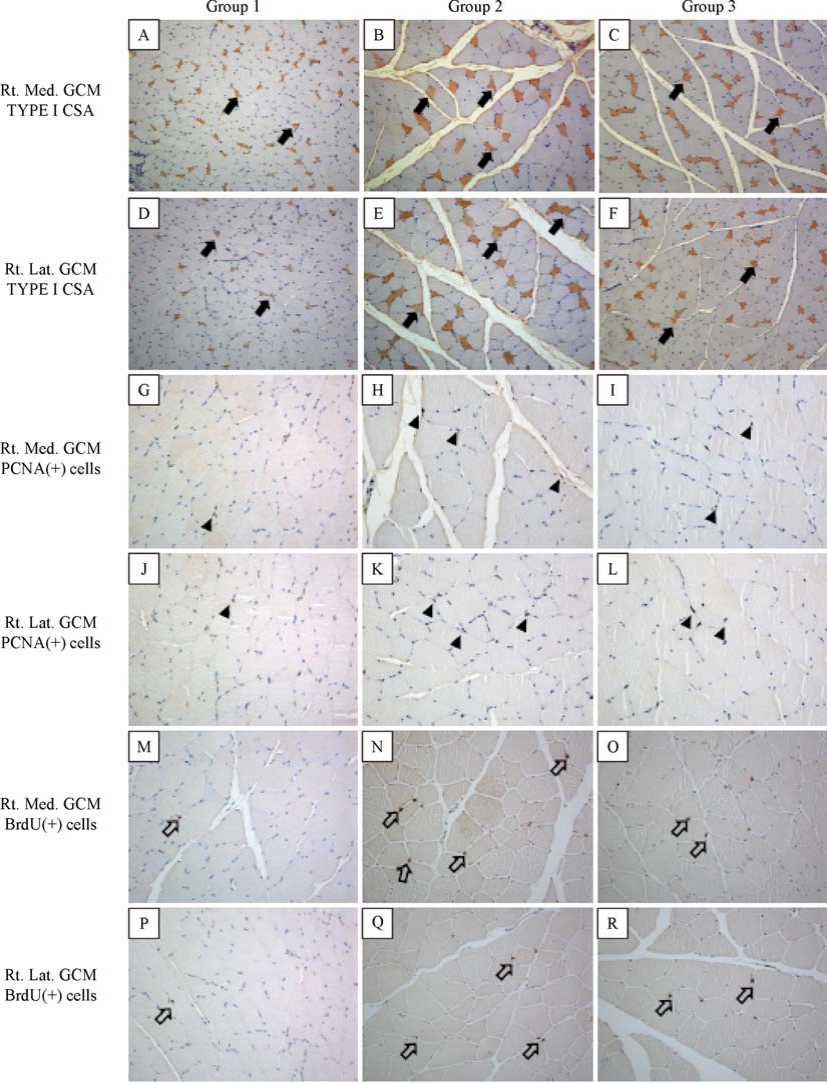
**Immunohistochemical findings of the right medial and lateral gastrocnemius muscles in group 1–3.** A–CF: Cross sectional areas of right medial and lateral gastrocnemius type I muscle fibres [monoclonal anti-myosin (skeletal, slow) antibody stain; ×100, arrows] were increased after microcurrent therapy. G–CL: PCNA positive cells were seen in the right medial and lateral gastrocnemius muscle fibres (arrow heads). PCNA positive cells or nuclei were significantly increased in group 2 as compared to group 1 and 3. M–CR: BrdU positive cells were seen in the right medial and lateral gastrocnemius muscle fibres (open arrows). The number of BrdU labelled cells or nuclei was increased after microcurrent therapy.

The histological parameters had significant differences among the three groups ( *P*<0.05, ***Table 2***, ***Fig. 2***). The mean CSAs of type 1 medial and lateral GCM muscle fibres in group 2 [(822.37±19.76) µm^2^, (870.43±21.57) µm^2^] and group 3 [(675.11±16.91) µm^2^, (684.06±32.80) µm^2^] were significantly greater than those in group 1 [(260.46±17.86) µm^2^, (262.51±14.31) µm^2^], respectively ( *P*<0.05, ***Table 2***). The mean CSAs of type 1 medial and lateral GCM muscle fibres in group 2 were significantly higher than those in group 3 ( *P*<0.05, ***Table 2***). The mean CSAs of type 1 medial and lateral GCM muscle fibres in group 2 were the largest CSAs among three groups ( *P*<0.05, ***Table 2***).

**Tab.2:** Comparison of the cross sectional area among three groups

Group	Rt. GCM type 1	
	Medial	Lateral
Group 1 ( *n*=5)	260.46±17.86^ a)^	262.51±14.31^ a)^
Group 2 ( *n*=5)	822.37±19.76^ b)^	870.43±21.57 ^b)^
Group 3 ( *n*=5)	675.11±16.91^ c)^	684.06±32.80^ c)^

Values are presented mean±standard error. Group 1: IC for 2 weeks and sham MT for 2 weeks after CR; Group 2: IC for 2 weeks and MT (25 μA for 2 weeks after CR; Group 3: IC for 2 weeks and MT (5,000 μAfor 2 weeks after CR; IC: immobilization by cast; MT: microcurrent therapy; CR: cast removal; CMAP: compound muscle action potential; GCM: gastrocnemius muscle; US: ultrasound; a), b), c): Any two means in the same row with different letters represent a significant difference at *P*<0.05, One Way ANOVA, post-hoc Tukey test.

The PCNA ratios (%) of type 1 medial and lateral GCM muscle fibres in group 2 (0.190±0.039, 0.189± 0.045) and group 3 (0.145±0.024, 0.138±0.016) were significantly higher than that in group 1 (0.070±0.018, 0.077±0.022) ( *P*<0.05, ***Table 3***). The PCNA ratio (%) of type 1 medial and lateral GCM muscle fibres in group 2 was significantly higher than that in group 3 ( *P*<0.05, ***Table 3***). The PCNA ratio (%) of type 1 medial and lateral GCM muscle fibres in group 2 was the highest among the three groups ( *P*<0.05, ***Table 3***).

**Tab.3:** Comparison of PCNA and BrdU ratio in medial GCM and lateral GCM among three groups.

Group	PCNA ratio		BrdU ratio	
	Medial GCM	Lateral GCM	Medial GCM	Lateral GCM
Group 1 ( *n*=5)	0.070±0.018^a)^	0.077±0.022^a)^	0.050±0.014^a)^	0.046±0.022^a)^
Group 2 ( *n*=5)	0.190±0.039^b)^	0.189±0.045^b)^	0.096±0.011^b)^	0.090±0.009^b)^
Group 3 ( *n*=5)	0.145±0.024^c)^	0.138±0.016^c)^	0.075±0.015^c)^	0.071±0014^c)^

Values are presented mean±standard error. Group 1: IC for 2 weeks and sham MT for 2 weeks after CR; Group 2: IC for 2 weeks and MT (25 μA for 2 weeks after CR; Group 3: IC for 2 weeks and MT (5,000 μAfor 2 weeks after CR; IC: immobilization by cast; MT: microcurrent therapy; CR: cast removal; CMAP: compound muscle action potential; GCM: gastrocnemius muscle; US: ultrasound; a), b), c: Any two means in the same row with different letters represent a significant difference at *P*<0.05, One Way ANOVA, post-hoc Tukey test.

The BrdU ratios (%) of type 1 medial and lateral GCM muscle fibres in group 2 (0.096±0.011, 0.090±0.009) and group 3 (0.075±0.015, 0.071± 0.014) were significantly higher than that in group 1 (0.050±0.014, 0.046±0.022) ( *P*<0.05, ***Table 3***). The BrdU ratio (%) of type 1 medial and lateral GCM muscle fibres in group 2 was significantly higher than that in group 3 ( *P*<0.05, ***Table 3***). The BrdU ratio (%) of type 1 medial and lateral GCM muscle fibres in group 2 was the highest among the three groups ( *P*<0.05, ***Table 3***).

## Discussion

The most significant finding of the present study is the greater regenerative effect seen in atrophied GCM muscle in group 2 than in groups 1 and 3, suggesting that low-intensity (25 μA) MT could minimize the detrimental effects induced by 2-week IC as compared to no MT or high-intensity (5,000 μA) MT. This result is supported by clinical and electrophysiologic imaging (calf circumference, CMAP of the tibial nerve, and GCM muscle thickness) and histological parameters (CSA of the type 1 muscle fibres, PCNA ratio, and BrdU ratio). There has been no previous study to compare the regenerative effects of different MT intensities on atrophied skeletal muscles. The current study reveals that low-intensity MT is more effective than high-intensity MT in treating skeletal muscle atrophy induced by IC.

Similar to other electrotherapies, the therapeutic effect of MT is also intensity-dependent. Previous studies have demonstrated that low-intensity electrotherapy improves the healing of damaged tendons and ligaments^[14,20–23]^. Dunn demonstrated the growth of fibroblasts in the collagen matrix in an experimental skin wound of the guinea pig^[23]^. The electric currents used in the experiment varied from 20 to 100 μA with the maximum fibroblast-growth response observed near the cathode. A recent study compared the effectiveness of MT with two different electric current intensities (50 μA *vs*. 500 μA), and suggested that a peak current intensity of 50 µA was more effective than 500 µA in alleviating symptoms and promoting tendon normalization in chronic tennis elbow^[24]^. When an intensity of 100–500 μA was applied in treating muscle damage, the healing processes (including amino acid transport, triphosphate generation, and protein synthesis) were boosted by 30%–40% above the control level^[14]^. On the contrary, when the intensity exceeded 1,000 μA, these bio-stimulatory effects were reversed.

In a previous study, when electric currents with 10–50 µA amplitude were used to treat muscle-related problems, different microcurrent machines were used^[25]^. Previous studies demonstrated that MT with low amperage electric current of<500 μA could reduce the severity of muscle symptoms^[26–28]^. These electric current intensities are within the therapeutic range, which is optimum for ATP generation. These results are consistent with the findings of the current study and suggest that low-amperage electric currents can more effectively improve skeletal muscle regeneration than high-amperage electric currents.

In the present study, we found that the number of PCNA and BrdU-positive satellite cells in the atrophied GCM muscle had a significant increase after low-intensity MT compared to high-intensity MT. Although the parameters of MT (8 Hz, 25 μA, 5,000 μA) used in this study were different from those used the in the previous studies, our findings are in agreement with those of earlier studies, in which the electrical stimulation (2–20 Hz, 0.5–20 mA) yielded an effective stimulus to rescue the loss of satellite cells in disused muscle atrophy in mice^[29]^, and a similar effect was found on muscle satellite cells with other electrical stimulation parameters (0.3 Hz and 10 μA)^[16]^. In particular, the mitotic activity of satellite cells (myogenic precursor cells) was the first observable phenomenon after the 2-week immobilisation followed by 4 weeks of remobilisation and coincided with the recovery of muscle fibres^[19,27]^.

Notably, the results of the present study showed that, compared with the effect of high-intensity MT, low-intensity MT can remarkably facilitate the regeneration of immobilised skeletal muscles through stimulating the proliferative potential of muscle satellite cells, instead of simply influencing the remobilisation of skeletal muscles. In addition, no adverse effects or untoward events were observed during the study, because MT worked at the microampere level and mimicked the electrical intensity found in the living tissue^[29–30]^.

Our study had a few limitations. First, since we did not subject the rabbits to any exercise regime, further study is needed to evaluate the combined effects of MT and different types of exercise (aerobic or anaerobic). Second, considering that this study was conducted in a relatively short time period, it would be necessary to assess the long-term effects of MT. Third, no control group was included in our present study. Last, additional studies are needed to evaluate the effects of MT of various frequencies and durations (<30 minutes or>several hours) for optimal results.

In conclusion, our results showed that low-intensity MT promotes regeneration in atrophied GCM muscle in a more effective way than high-intensity MT in the rabbit model. Therefore, compared with high intensity MT, low-intensity MT maybe more effective in treating the clinical conditions related to skeletal muscle atrophy.
